# Adaptation of soil nitrifiers to very low nitrogen level jeopardizes the efficiency of chemical fertilization in west african moist savannas

**DOI:** 10.1038/s41598-017-10185-5

**Published:** 2017-08-31

**Authors:** Féline L. Assémien, Thomas Pommier, Jean T. Gonnety, Jonathan Gervaix, Xavier Le Roux

**Affiliations:** 1 INRA, CNRS, Université Lyon 1, Université de Lyon, Laboratoire d’Ecologie Microbienne, UMR 1418 LEM, UMR 5557 CNRS, 69622 Villeurbanne Cedex, France; 2 Université Nangui Abrogoua, Unité de formation et de Recherche des Sciences et Technologies des Aliments, Laboratoire de Biocatalyse et des Bioprocédés, 02 BP 801 Abidjan 02, Ivory Coast

## Abstract

The moist savanna zone covers 0.5 × 10^6^ km^2^ in West Africa and is characterized by very low soil N levels limiting primary production, but the ecology of nitrifiers in these (agro)ecosystems is largely unknown. We compared the effects of six agricultural practices on nitrifier activity, abundance and diversity at nine sites in central Ivory Coast. Treatments, including repeated fertilization with ammonium and urea, had no effect on nitrification and crop N status after 3 to 5 crop cycles. Nitrification was actually higher at low than medium ammonium level. The nitrifying community was always dominated by ammonia oxidizing archaea and *Nitrospira*. However, the abundances of ammonia oxidizing bacteria, AOB, and *Nitrobacter* increased with fertilization after 5 crop cycles. Several AOB populations, some affiliated to *Nitrosospira* strains with urease activity or adapted to fluctuating ammonium levels, emerged in fertilized plots, which was correlated to nitrifying community ability to benefit from fertilization. In these soils, dominant nitrifiers adapted to very low ammonium levels have to be replaced by high-N nitrifiers before fertilization can stimulate nitrification. Our results show that the delay required for this replacement is much longer than ever observed for other terrestrial ecosystems, i.e. > 5 crop cycles, and demonstrate for the first time that nitrifier characteristics jeopardize the efficiency of fertilization in moist savanna soils.

## Introduction

Nitrogen, N, limits primary productivity in many terrestrial ecosystems^1^ and its dynamics depends on key microbial activities such as N_2_ fixation, mineralisation, nitrification, denitrification and anaerobic ammonium oxidation. Nitrification is particularly important for soil fertility. It consists in the oxidation of ammonia to nitrite by ammonia oxidizing bacteria and archaea (AOB and AOA, respectively)^2, 3^, and the oxidation of nitrite to nitrate by nitrite oxidizing bacteria (NOB)^4, 5^. Nitrification plays a key role in determining how much and which forms of soil inorganic N are available for plants, and consequently in driving N losses from ecosystems through nitrate leaching^6^ and nitrogen oxide emission^7, 8^.

Soil N-availability influences the abundance and the composition of nitrifying groups. Indeed, within soil ammonia-oxidizers, AOA and AOB occupy to some extent different ecological niches^9–11^. AOA are generally favored by low ammonium levels^12–14^ and different studies reported that N addition does not influence or decrease AOA abundance in grassland soils^15–19^ (but see refs 20, 21). In contrast, AOB exhibit high activity in environments with high ammonium availability^22, 23^. Besides, two major genera of NOB are present in soil, i.e. *Nitrobacter* and *Nitrospira* ^24, 25^, although NOB are actually more diverse and their ecology is more complex than previously estimated^26^. Functional diversity exists within each of the two groups, but *Nitrospira* are generally assumed to thrive in low N levels, whereas *Nitrobacter* outcompete *Nitrospira* under high N levels^27–29^. These ecological traits are consistent with the observed responses of soil NOB to environmental changes and agricultural or forestry practices^5, 19, 29, 30^. Accordingly, the composition of nitrifying communities can influence the capacity of soil to respond to environmental changes, such as changes in land use or management. For instance, in an experiment mimicking a reversion of grazing regime through urea application and plant clipping in grasslands from central France, Le Roux *et al*.^31^ observed a preliminary shift in the community structure of ammonia-oxidizers over a few months before a change in nitrification activity occurred. Similarly, Webster *et al*.^32^ reported that a change in AOB community structure –which occurred over a few weeks–, was required before enhancement of nitrification following sheep urine application on ungrazed grassland soils from the United Kingdom. This suggests that when the ammonia oxidizing communities are dominated by taxa sensitive to high ammonia concentration, a shift toward ammonia-tolerant populations is needed before nitrification can increase in response to increased N availability. These previous studies reported rather short time lags before high ammonia-tolerant nitrifiers can emerge as dominant community members (i.e. weeks to months), likely because the relatively N-poor soils studied harboured –although at low abundance– nitrifiers adapted to high-N microhabitats. Such time lags may thus have minor effects from an agronomic perspective. However, the time lag could be more important in terrestrial biomes with particularly N-poor soils where high-N microhabitats and nitrifiers adapted to high N are nearly absent. The choice of land management and fertilization practices should then consider the status of soil nitrifying communities, in particular when designing agricultural practices to improve N cycling and availability in very N-poor soils.

The moist savanna zone covers 0.5 10^6^ km^2^ in West Africa^33^ and is characterized by soils with particularly low total N concentrations (<0.1% in non cultivated areas under savanna grass cover for decades), and very low mineral N concentrations (<1 µg mineral N g^−1^ soil^7, 34, 35^). Based on field surveys, De Rham^36^ estimated the annual production of mineral N in moist savanna soils in Ivory Coast to amount 0.2 to 0.5 g N m^−2^ y^−1^, while Abbadie & Lensi^37^ reported very low ammonification rates (around 0.7 µg N-NH_4_^+^ g^−1^ soil day^−1^). Field trials in this savanna zone have demonstrated that N strongly limits primary production^38^. Due to the intense demographic pressure (i.e. 2.7% increase in local human populations between 1998 and 2007^39^), and given that most suitable areas are cultivated, the pressure on cultivated surfaces is increasing, and farmers strive to choose adequate agricultural practices for obtaining economically viable crop yields on such poor soils. Traditionally, farmers in this area use the slash-and-burn practice, *i.e*. they burn the existing vegetation from savannas or fallows, thus enriching the soil with ashes, cultivate these soils during a few years and then let the plots uncultivated to exploit other areas. However, this traditional practice is damaging soil quality in particular when cultivation cycles become more frequent^40^. Chemical fertilization, *i.e*. the addition of ammonium and/or urea as widely used in this area, has thus been increasingly used to improve N availability and potentially increase crop yield. The use of nitrate as fertilizer is often avoided because it can be leached more easily. An alternative practice is the use of mulching: after harvest during the crop rotation, farmers cut the standing biomass and let it on the ground to create a mulch over the soil. However, the effects of these different post-fallow agricultural practices on nitrification and nitrifiers remain unknown for African savannas. In particular, when cultivating crop varieties that partly or mainly depend on soil nitrate, the efficiency of chemical N fertilization could be jeopardized, at least initially, if the soil nitrifying community is unable to perform well under the higher levels of ammonium and urea induced by chemical fertilization. The mulching technique recently introduced in the moist savanna zone, using crop rotations with or without the insertion of legumes, may allow a more progressive increase in the soil organic matter and N availability levels.

The objective of this study was to compare and understand the effects of 6 different agricultural practices applied over 2.5 years on nitrification and nitrifiers in such N-poor soils: (1) slash-and-burn with continuous maize rotation; (2) chemical fertilization with continuous maize rotation; (3) mulching with continuous maize rotation; (4) mulching with maize-soya rotation; (5) mulching with maize-bean rotation; and (6) bare soil with continuous maize rotation. A same field trial with 6 plots corresponding to these 6 practices was replicated 9 times across a small landscape in central Ivory Coast (see Figs S1 and S2). We measured key soil environmental variables (moisture, ammonium, pH), nitrification, and the abundances of key nitrifying microbial groups (AOB, AOA, *Nitrobacter* and *Nitrospira*) for each of the 54 plots after 3, 4 and 5 crop cycles. After 5 crop cycles, we also compared the sensitivity of nitrification to two levels of ammonium (low: 2 µg N-NH_4_^+^ g^−1^ soil; and medium: 10 µg N-NH_4_^+^ g^−1^ soil) and the AOB community composition between the different treatments. We hypothesized that (i) the nitrifying communities in the soils from the moist savanna zone result from an adaptation to very low N levels, so that fertilization can negatively influence nitrification in these soils; (ii) the stimulation of nitrification by chemical fertilization first requires profound changes in the nitrifier community, *i.e*. the replacement of the dominant nitrifiers adapted to very low N levels by nitrifiers adapted to higher N levels; and (iii) the time lag associated to the changes in the nitrifier community after fertilization inception or use of legumes may be so important that it would have agronomic implications (i.e. the time lag would correspond to one or several crop cycles). We also assumed that the mulching technique with legumes inserted in crop rotations may allow a more progressive increase in soil N availability to nitrifiers and a more progressive shift in the soil nitrifier community, which may be less costly to farmers and more adequate than chemical fertilization for these soils. Overall, our results demonstrate for the first time the adaptation of nitrifiers to very low N levels, and how this can jeopardize the efficiency of fertilization in these moist savanna soils

## Results

### Soil environmental parameters

Sampling date significantly influenced soil moisture (p < 0.0001) while the treatment and time X treatment interaction effects were not significant (p = 0.86 and 0.97, respectively). Soil moisture was highest (13–14%) at the second sampling date, intermediate (7–8%) at the first date, and lowest (4–5%) at the third sampling date (Fig. S3). At each sampling date, soil moisture was not significantly influenced by treatments (p = 0.99, 0.94 and 0.33 after 3, 4 and 5 crop cycles, respectively). Similarly, sampling date significantly influenced soil pH (p = 0.002) while the treatment and time X treatment interaction effects were not significant (p = 0.82 and 0.93, respectively). However, soil pH was always in the range 6.5 to 6.7 (Fig. S4).

Sampling date significantly influenced soil ammonium concentration (p < 0.0001) while the treatment and time X treatment interaction effects were not significant (p = 0.60 and 0.74, respectively). At each of the three sampling dates, no significant treatment effect was observed on soil ammonium concentration, even for plots receiving ammonium plus urea fertilization (Fig. S5). Ammonium concentration was always lower than 0.8 and 0.4 µg N-NH_4_^+^ g^−1^ soil at the first and second dates, respectively, and was highest (1.5 to 3.0 µg N-NH_4_^+^ g^−1^ soil) at the last sampling date (Fig. S5). After 5 crop cycles, treatment effect on soil nitrate concentration was not significant with values ranging from 1.5 to 2.0 µg N-NO_3_^−^ g^−1^ soil for all treatments (Fig. S6).

After 3 and 5 crop cycles, no significant treatment effect was observed on soil organic concentration and the proxy of microbial biomass, i.e. dehydrogenase activity (Fig. S7). In contrast, the effects of sampling date, treatment and time X treatment interaction on total soil nitrogen were all significant (p < 0.0001, 0.016 and 0.02, respectively). Actually total soil nitrogen was not influenced by treatment after 3 crop cycles, but became higher in fertilized plots and plots with mulching and a maize-bean rotation as compared to control plots after 5 crop cycles (Fig. S7).

### Nitrification and sensitivity to ammonium concentration

**Figure 1:**
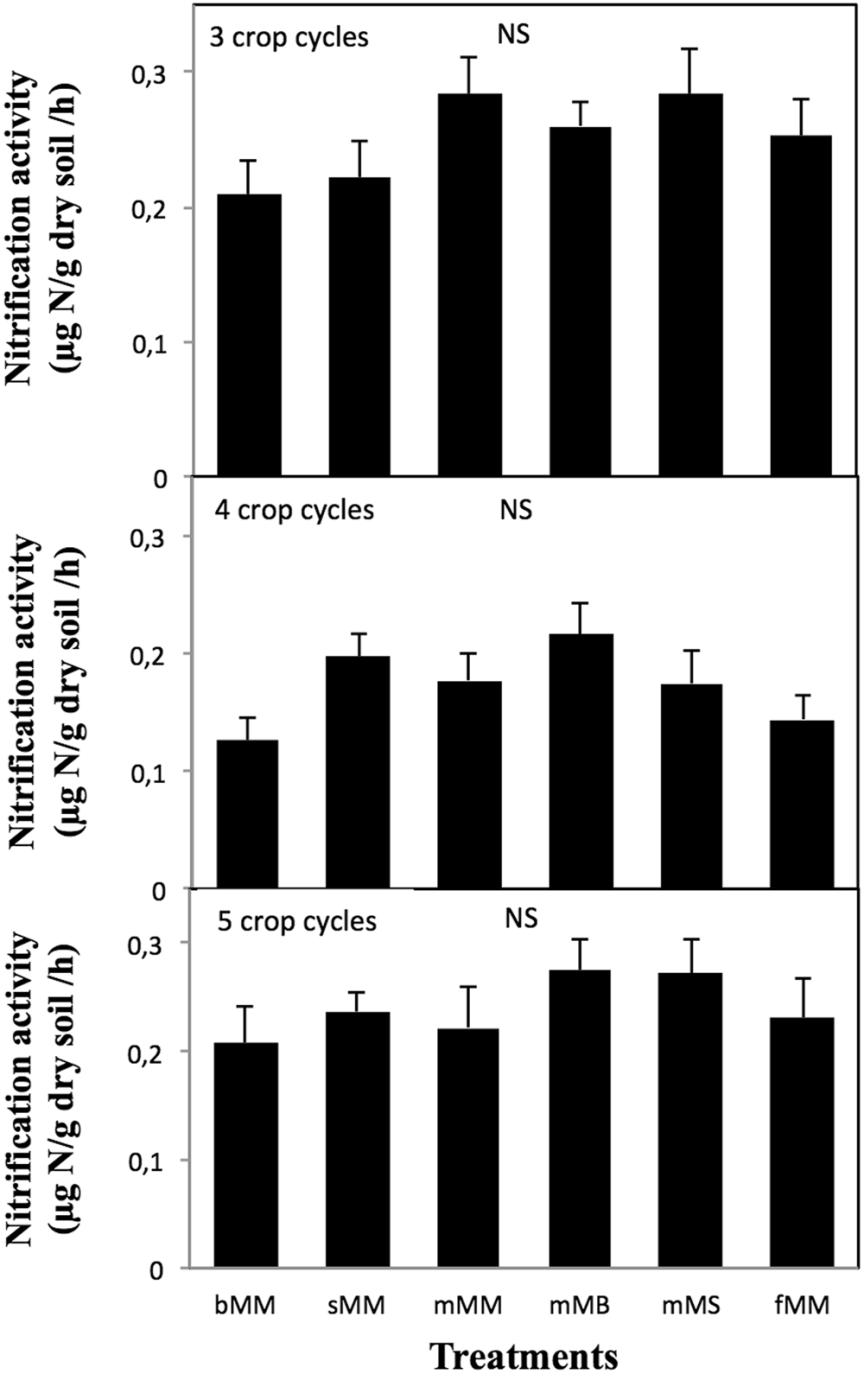
Nitrification activity in soils under the different treatments after 3, 4 and 5 crop cycles. Nitrification was measured at an ammonium concentration of 2 ppm. Six treatments were studied: bMM, maize-maize rotation on bare soil; sMM, maize-maize rotation with the slash-and-burn practice; mMM, maize-maize rotation with mulching; mMB, maize-bean rotation with mulching; mMS, maize-soya rotation with mulching; fMM, maize-maize rotation with chemical fertilization. Error bars are standard errors (n = 9). NS indicates a lack of significant difference between treatments.

Nitrification ranged from 0.12 to 0.28 µg N g^−1^ soil h^−1^. Sampling date only significantly influenced nitrification (p < 0.0001), nitrification being lowest after 4 crop cycles (Fig. 1). Nitrification was not significantly influenced by treatments, whatever the sampling date (p = 0.27, 0.09 and 0.71 after 3, 4 and 5 crop cycles, respectively) (Fig. 1). In particular, nitrification remained similar in fertilized plots and in other plots, even after 5 crop cycles – that is 2.5 years after treatment inception (Fig. 1).

The percentage of change of nitrification at medium (10 µg N-NH_4_^+^ g^−1^ soil) compared to low (2 µg N-NH_4_^+^ g^−1^ soil) levels of ammonium level (i.e. 100 * (Nitrif_10_ − Nitrif_2_)/Nitrif_2_), was significantly (p = 0.050) influenced by treatments at the last sampling date. It ranged from + 3% for fertilized plots (*i.e*. nitrification similar for both ammonium levels), to −9.3% and −17.8% for plots with mulching and a maize-maize and maize-bean rotation, respectively, down to −22.6%, −25.5% and −26.8% for bMM, sMM and mMS plots, respectively.

**Figure 2:**
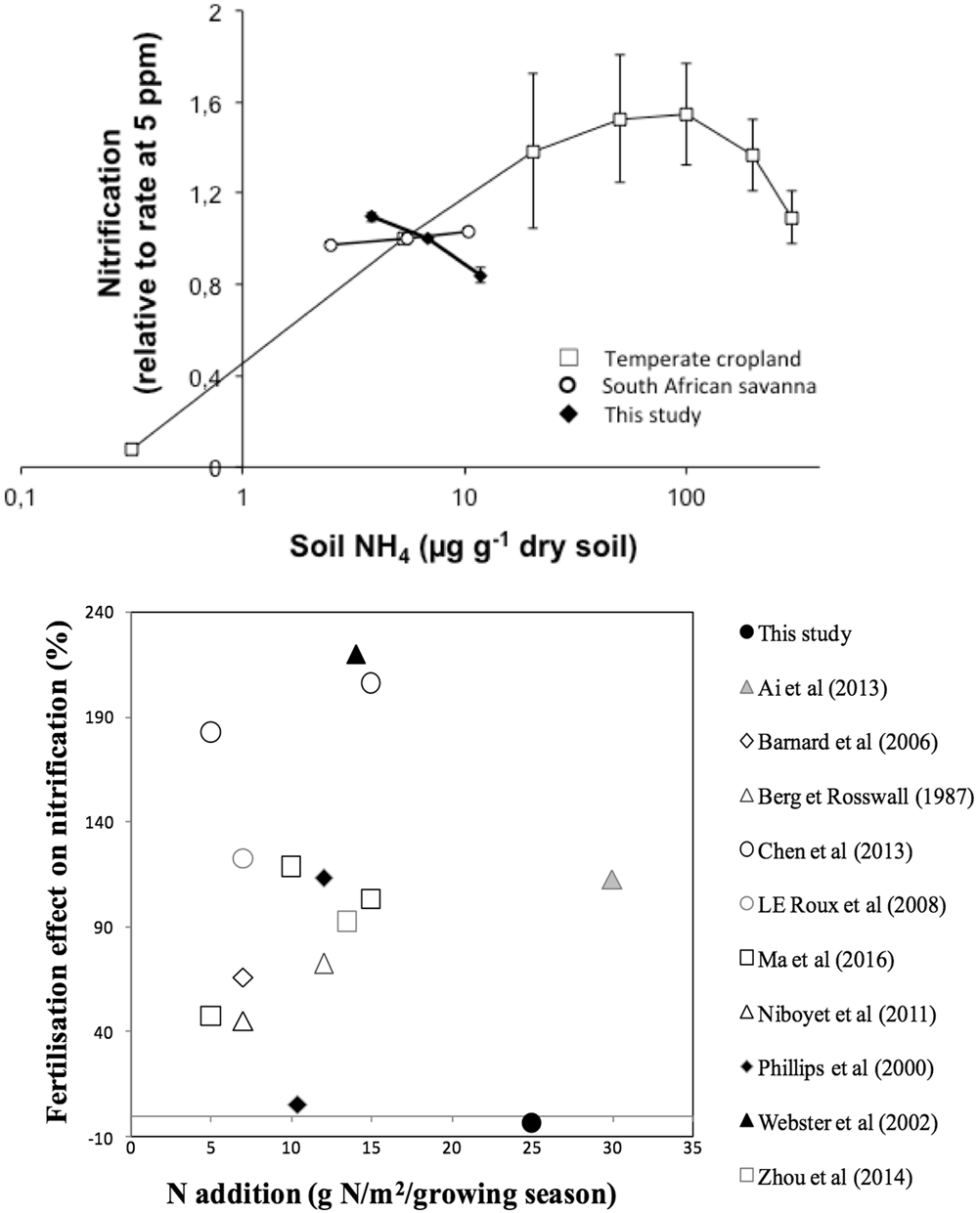
(Top) Comparison of the response of nitrification to soil ammonium concentration between a temperate cropland soil, a south African savanna soil, and the studied soil. For each soil, the nitrification rate is expressed as compared to the rate observed for 5 ppm ammonium (a linear extrapolation between 2 and 10 ppm was used for the studied soil). Error bars are standard errors and are hidden by the symbol for the South African savanna soil. (Bottom) Comparison of N fertilization effect on nitrification reported for different croplands and grasslands. The effect of N fertilization on nitrification observed in the present study is compared with effects reported in the literature for a range of cropping and grassland systems. For each study, the fertilization effect is expressed as the percentage change compared to nitrification in control, unfertilized plots, i.e. 100 * (Nitrif_Fertilized_ − Nitrif_Unfertilized_)/Nitrif_Unfertilized_. Each symbol corresponds to a published study (see Table S1 for details).

We compared the response of nitrification to ammonium level observed here with the response obtained for a temperate and fertilized cropland soil and a South African savanna soil. For the temperate and fertilized cropland soil, nitrification increased with increasing ammonium concentration up to 50–100 µg N-NH_4_^+^ g^−1^ soil and decreased only for ammonium concentrations higher than 100 µg N-NH_4_^+^ g^−1^ soil (Fig. 2). For the South African savanna soil, nitrification weakly increased for increasing ammonium concentration in the range 2 to 10 µg N-NH_4_^+^ g^−1^ soil (Fig. 2).

### Comparison with fertilization effects on nitrification reported in previous studies

The result of the literature survey for fertilizer effects on nitrification in cropping and grassland systems (Fig. 2; Table S1) shows that the lack of stimulation of nitrification by fertilization observed in our study (*i.e*. no effect after 2.5 years, despite inputs equivalent to 25 g N m^−2^ growing season^−1^) is rather unusual. Indeed, nitrification tended to be strongly stimulated by N fertilization in both grassland and cropping systems. The reported stimulation levels ranged from +45% to +182% for fertilizer inputs ranging from 5 to 7 g N m^−2^ growing season^−1^; and from +72% to +220% for fertilizer input from 10 to 30 g N m^−2^ growing season^−1^, except for one of the two values reported by Philipps *et al*. (2000) (Fig. 2; Table S1).

### Ammonia- and nitrite-oxidizer abundances

**Figure 3:**
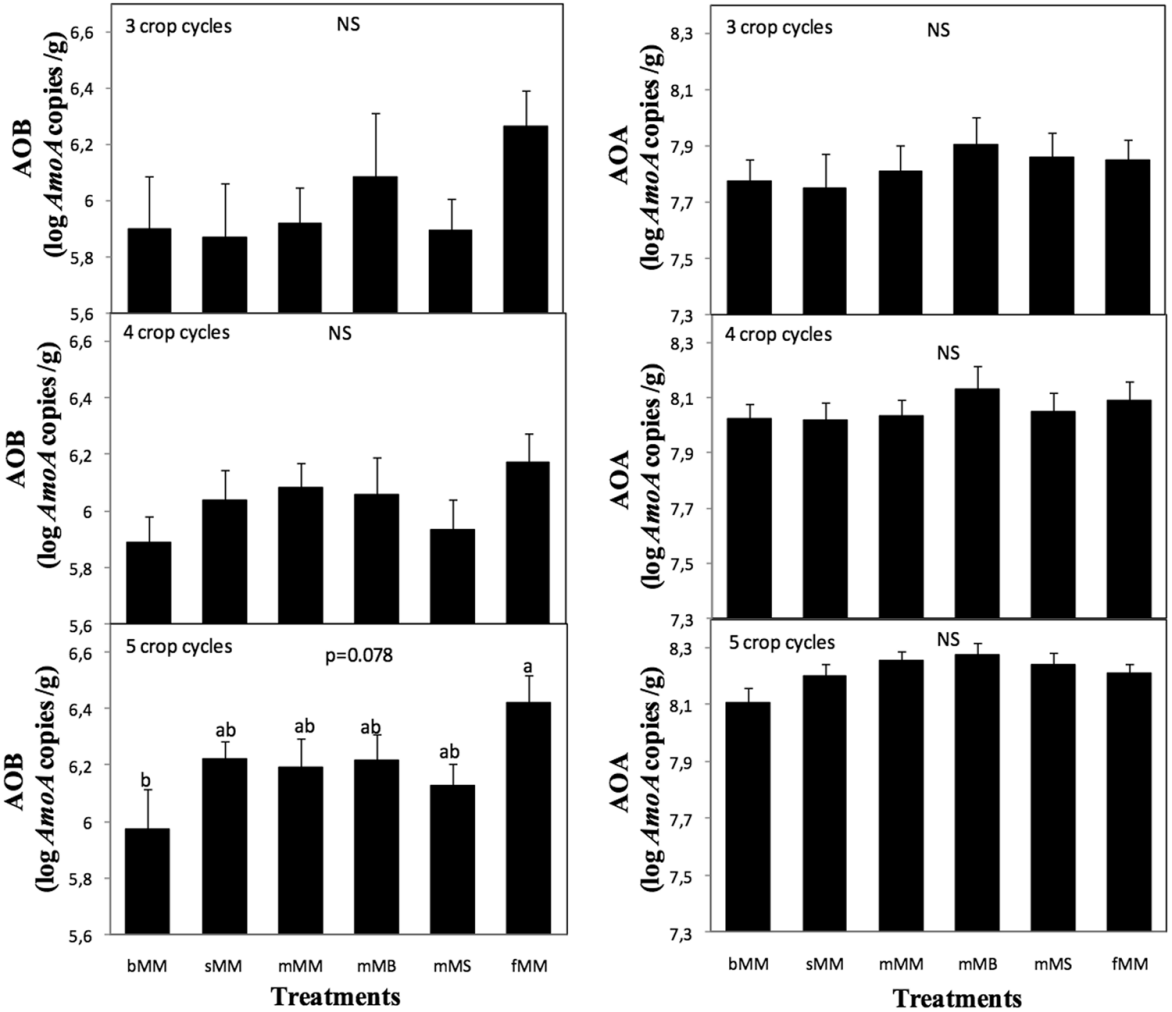
Abundances of ammonia oxidizers according to treatment. Abundances are presented for (Left) ammonia oxidizing bacteria, AOB, and (Right) ammonia oxidizing archaea, AOA, after 3, 4 and 5 crop cycles. Treatment acronyms are as in Fig. 1. Error bars are standard errors (n = 9). NS indicates lack of significant difference (p always > 0.1 here) and the marginally significant (p = 0.078) treatment effect on AOB after 5 cycles is indicated.

The average abundance of AOA was around 10^8^ *amoA* copies g^−1^ soil. Sampling date significantly influenced AOA abundance (p < 0.0001) while the effects of treatment and time X treatment interaction were not significant (p = 0.23 and 0.99, respectively), indicating that treatments had no effect on AOA abundance whatever the sampling date (Fig. 3). The average abundance of AOB was much lower (around 10^6^ *amoA* copies g^−1^ soil). Sampling date and treatments significantly influenced AOB abundance (p = 0.013 and 0.012, respectively) while the time X treatment interaction effect was not significant (p = 0.99). Focusing on each date however, AOB abundance tended to be highest in fertilized plots (p = 0.078) only at the last sampling date (Fig. 3).

**Figure 4:**
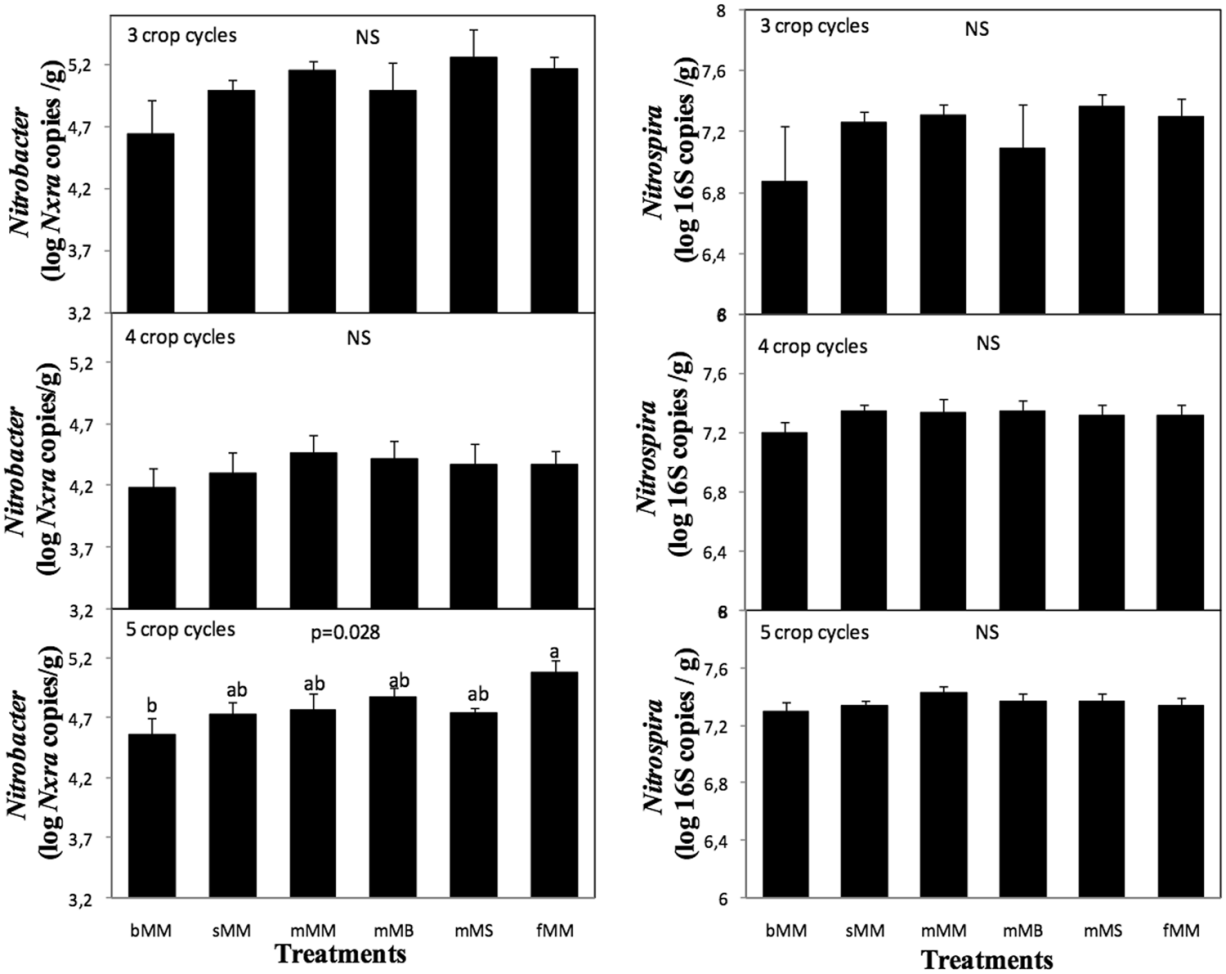
Abundances of nitrite oxidizers according to treatment. Abundances are presented for (Left) *Nitrobacter* and (Right) *Nitrospira* after 3, 4 and 5 crop cycles. Treatment acronyms are as in Fig. 1. Error bars are standard errors (n = 9). Different letters indicate significant differences between treatments; NS indicates lack of significant difference (p always > 0.1 here).

The abundance of *Nitrospira* was always around 2 × 10^7^ *16 S* copies g^−1^ soil and was not influenced by treatment, sampling time, nor time X treatment interaction (p = 0.10, 0.24 and 0.88, respectively) (Fig. 4). The average abundance of *Nitrobacter* was around 5 × 10^4^ *nxrA* copies g^−1^ soil. Sampling date and treatment significantly influenced *Nitrobacter* abundance (p < 0.0001 and 0.019, respectively) while the time X treatment interaction effect was not significant (p = 0.84). However, focusing on each date, *Nitrobacter* abundance was significantly (p = 0.028) highest in fertilized plots and lowest in the bare soil plots, only at the last sampling date (Fig. 4).

The correlations between the abundances of the different nitrifier groups changed with time (Fig. S8). Whereas no significant correlation was observed between *Nitrobacter* and AOB abundances after 3 crop cycles, the correlation became significant after 4 cycles and was very strong after 5 cycles (Fig. S8). Similarly, no significant correlation was observed between *Nitrospira* and AOA abundances after 3 and 4 crop cycles, but the correlation became significant after 5 cycles (Fig. S8). In contrast, a strong correlation was observed between *Nitrobacter* and *Nitrospira* abundances after 3 crop cycles, but the correlation became weaker after 4 cycles and was not significant anymore after 5 cycles (Fig. S8). The correlation between AOB and AOA abundances was never significant (not shown).

### Diversity and phylogeny of the soil ammonia oxidizing bacteria

The overall AOB community structure quantified after 5 crop cycles was not significantly influenced by treatment (P = 0.93; R = 0.045; see Fig. S9) and was significantly correlated to soil moisture (P = 0.006; ρ = 0.171) and marginally correlated to ammonium concentration (P = 0.088; R = 0.102). The changes in nitrification were correlated to changes in the AOB community structure (P = 0.001; R = 0.182).

**Figure 5:**
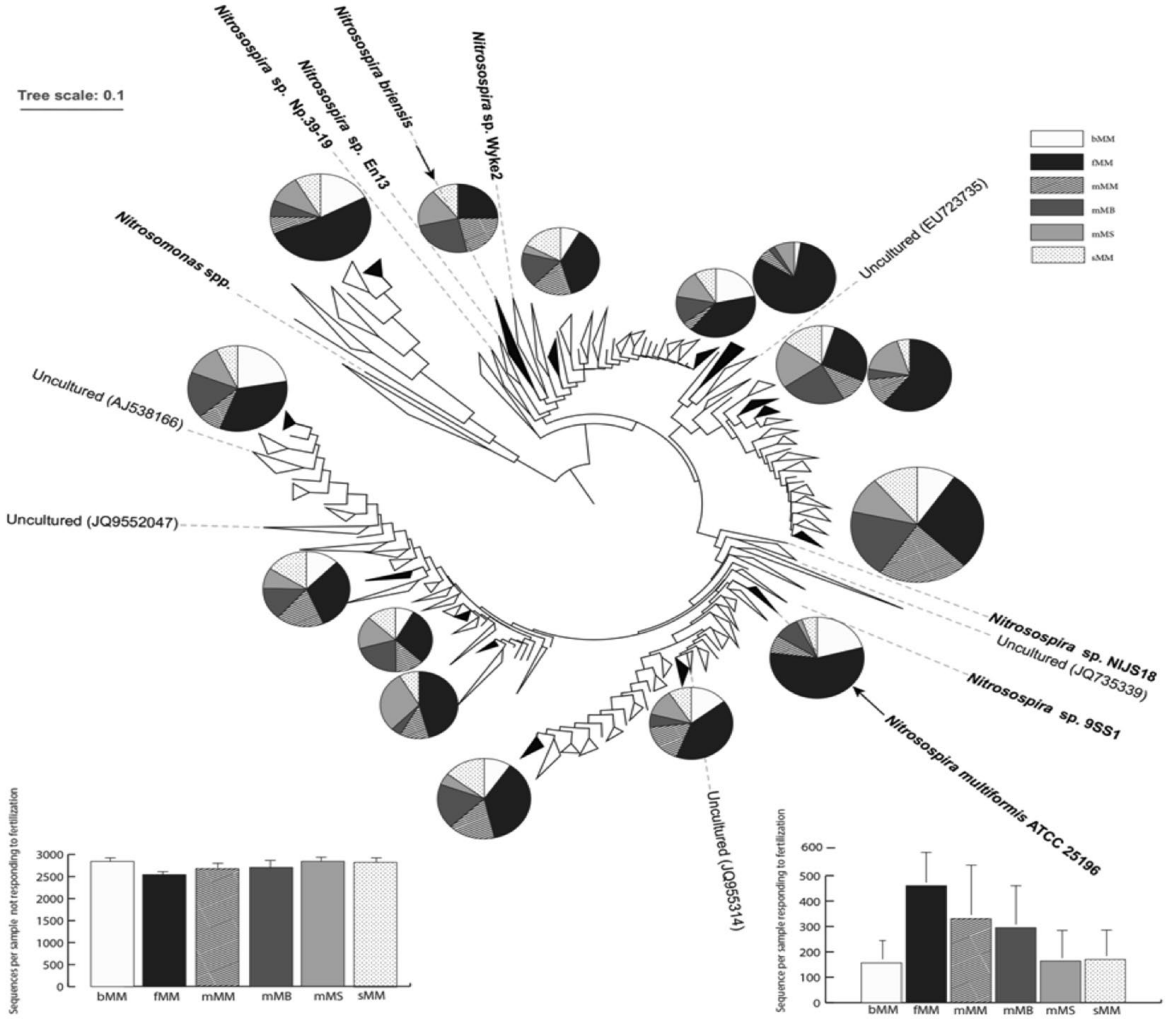
Phylogenetic relationships among AOB *amoA* sequences retrieved from soils under the 6 treatments after 5 crop cycles. Black and white clusters correspond to clusters of OTUs whose relative abundance increased and did not change or decreased in response to fertilization, respectively. Disks indicate the distribution of *amoA* sequences among the six treatments for each black cluster (disk size corresponds to sequence abundance in log scale). Bold and plain names indicate the location of *amoA* sequences of cultivated strains and reference sequences, respectively. The insets present the total number per sample of sequences belonging to (Bottom Left) white clusters, and (Bottom Right) black clusters according to treatment, with mean values and standard errors (n = 9).

The phylogenetic tree obtained for the AOB *amoA* sequences retrieved from the studied soils is presented in Fig. 5. The relative abundance of *amoA* sequences from 17 sub-clusters increased in response to fertilization (Fig. 5), these OTUs representing over 17% of the AOB community in fertilized plots, and only 5.8% and 6.7% in bare soil and slash-and-burn plots, respectively. *AmoA* sequences from three of these 17 clusters were affiliated to known AOB (Fig. 5): one cluster was affiliated to *Nitrosospira multiformis*, one to *Nitrosospira briensis*, and one to an uncultivated *Nitrosospira* sp. previously identified by He *et al*.^41^ in an agri-udic ferrosol under a subtropical climate in China.

### Relationship between the sensitivity of nitrification to ammonium level and the composition of the nitrifying community

**Figure 6:**
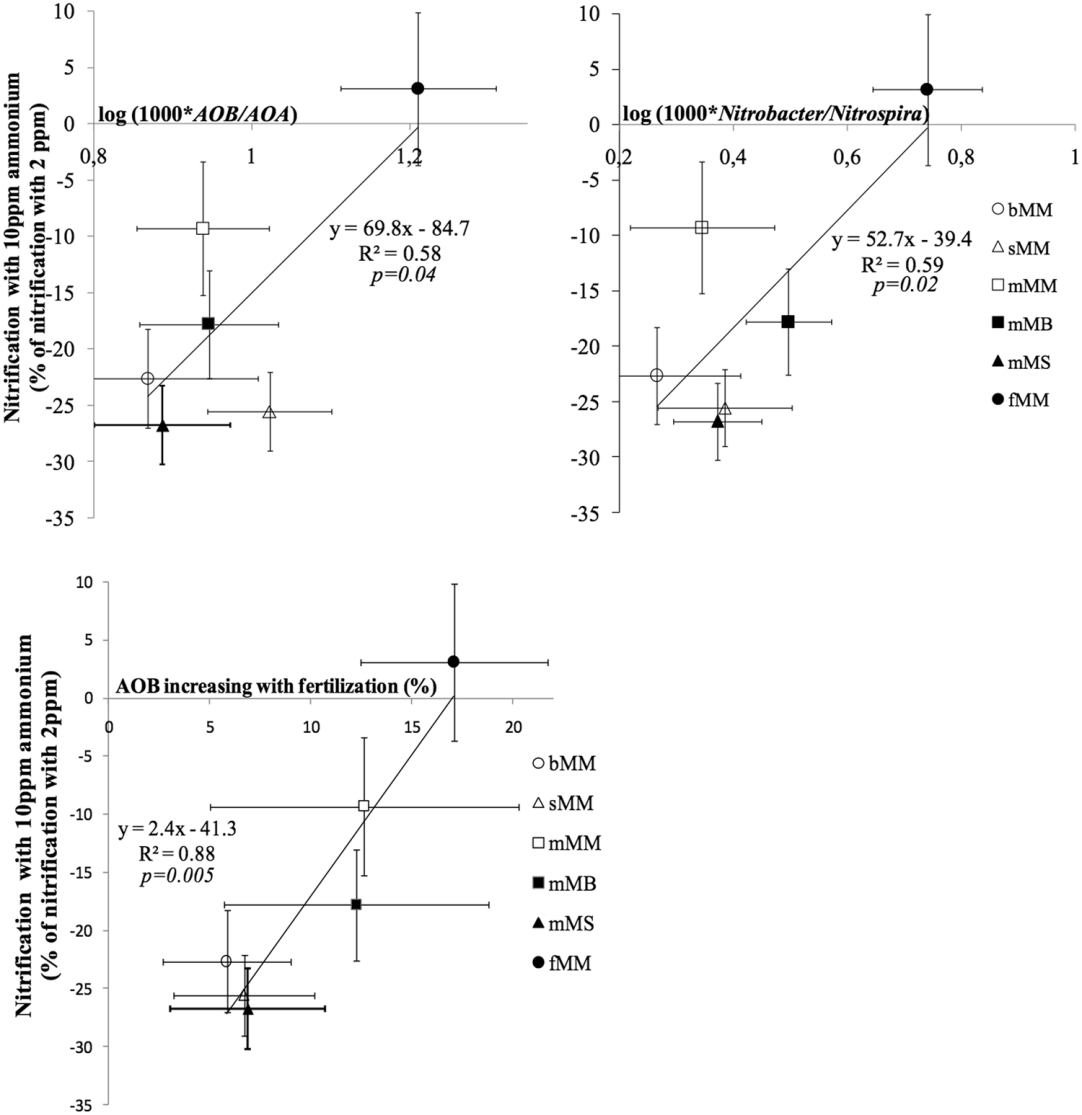
Sensitivity of nitrification to medium as compared to low ammonium levels is related to the composition of the nitrifying community. The percentage of change in nitrification measured under 10 ppm as compared to 2 ppm ammonium is related to (Top-Left) the ratio of the abundances of ammonia oxidizing bacteria to ammonia oxidizing archaea, AOB/AOA, (Top-Right) the *Nitrobacter*-to-*Nitrospira* ratio, and (Bottom-Left) the relative abundance of AOB populations that increased in response to fertilization. Treatment acronyms are as in Fig. 1.

The percentage of change of nitrification at 10 ppm as compared to 2 ppm was significantly (p = 0.044) correlated to the AOB/AOA ratio: the higher the AOB/AOA ratio, the better the nitrification at medium as compared to low ammonium (Fig. 6). A significant correlation (p = 0.018) was also observed between the percentage of change of nitrification and the *Nitrobacter*/*Nitrospira* ratio (Fig. 6). Moreover, the percentage of change of nitrification measured for 10 ppm as compared to 2 ppm ammonium was highly correlated (p = 0.0049, R^2^ = 0.88) to the sum of the relative abundances of all the *amoA* clusters promoted by fertilization (Fig. 6-Bottom).

### Mineral N uptake capacities of the maize cultivar, and N concentration in crop leaves, stems, and roots

N uptake rate by the maize cultivar was higher at 17 ppm than 6 ppm total mineral N (Fig. S10). At 6 ppm, NO_3_^−^ and NH_4_^+^ uptake rates were 0.035 and 0.004 mg N h^−1^ g^−1^ root, respectively, whereas at 9 ppm these rates were 0.057 and 0.009 mg N h^−1^ g^−1^ root, respectively (Fig. S10). The ratio of nitrate-to-ammonium uptake rates was thus 8.7 and 6.1 at the low and medium N concentration, respectively.

After 5 crop cycles, stem and root N concentrations ranged from 0.67 to 1.3%, and 0.95 to 1.4%, respectively, without any significant effect of treatments (p = 0.30 and p = 0.57, respectively) (Fig. S11). Leaf N concentration ranged from 1.3 to 2.6% and was significantly higher (p < 0.018) for beans (mMB treatment) than maize from the bare soil (bMM) and slash-and-burn (sMM) treatments. However, leaf N concentration did not differ between all the treatments with maize plants during the fifth crop cycle, i.e. bMM, sMM, mMM and fMM (Fig. S11).

## Discussion

Our results support the view that soils from the moist savanna zone in Ivory Coast have particularly low ammonium levels^42^. Indeed, except during very dry periods as for the last sampling date, the soil ammonium concentration in the cropped systems studied here was always lower than 0.8 μg N g^−1^ dry soil. This is consistent with the peculiar N cycling that characterizes soils from the moist savanna zone. First, these soils are very sandy with very low silt and clay contents (typically 14 and 7%, respectively, in the study area) and with very low cation exchange capacity, CEC (ca. 3.0 cmol kg^−1^ for non cultivated savanna soils), which does not favor the retention of nutrients. Although we did not measure soil CEC in the present study, previous studies for the same region of Ivory Coast reported low CEC values for maize crop soils^43^ (ca. 8 cmol kg^−1^) and for fallow soils^44^ (ca. 8 cmol kg^−1^), which can be compared to values ranging from 26 to 34 cmol kg^−1^ for typical silt-loam and clay-loam soils from the USA^45^. Second, intense disturbances like annual fires and the sub-equatorial climate favor N losses by biomass burning and N leaching^46^. Third, biological N_2_ fixation is extremely low in these soils, reducing the N inputs in the ecosystems^46^. These features explain that these savanna soils are among the vegetated soils with the lowest ammonium levels on earth^46^.

Although soil ammonium concentrations tended to be highest in fertilized and mulching plots, this trend was not significant. At the two first sampling dates (*i.e*. before the end of the crop cycle and during a wet period), ammonium concentrations remained lower than 0.8 μg-N g^−1^ soil even in fertilized plots despite the high amount of N fertilizer added at days 0 and 40 of each crop cycle. This can hardly be explained by volatilization of the N amended to the soil, as ammonia volatilization mainly occurs on alkaline soils^47, 48^ and the pH of the soils studied here ranged from 6.5 to 6.8. A more likely explanation is that ammonium/urea concentrations increased following each fertilization event but that there was a relatively rapid leaching of amended N due to the particularly low cation exchange capacity of these soils and high rainfall typical of the sub-equatorial climate. Further studies of N leaching from these soils shall be conducted. In addition, part of the added ammonium may have been assimilated and stored in crop roots and in the total microbial biomass, which is consistent with the observed increase in total soil nitrogen in fertilized plots after 5 crop cycles.

Consistently with the very low soil ammonium level, the nitrifying community in these soils was dominated by groups known to be mostly adapted to low N level. In particular, the ammonia oxidizing community was dominated by AOA (ca. 100-fold more abundant than AOB). Several eco-physiological studies have showed that AOB often have a greater fitness and greater nitrite production rate at higher ammonium level than AOA^49^ although functional diversity exists within each group. Moreover, *Nitrospira* were 300 to 1000-fold more abundant than *Nitrobacter* in these soils. This is likely explained by the niche differentiation between both groups, mostly regarding N availability^29^. Indeed, *Nitrospira* and *Nitrobacter* have low and high half-saturation constants for nitrite, respectively, and *Nitrospira* often outcompete *Nitrobacter* under conditions of low N availability^27, 50, 51^. This is also supported in studies on NOB dynamics in soils^5, 30^. The high dominance of the nitrifying community by AOA and *Nitrospira* is thus consistent with the very low ammonium concentrations in these soils, which has also been reported for South African savannas^52^.

Moreover, our results provide strong evidence of the nitrifier adaptation to very low N levels. Indeed, we observed a reduction down to −30% of nitrification measured at the medium (10 ppm) as compared to low (2 ppm) ammonium level for unfertilized plots. Such a strong decrease of the activity of the soil nitrifying community over this range of ammonium levels is clearly particular when compared to the nitrifier response to increased ammonium concentration observed for the temperate fertilized cropped soil (Fig. 2): in this case, the optimal ammonium concentration is 50–100 ppm, highlighting nitrifier adaptation to high N levels. For the mesic savanna soil from South Africa, nitrification only weakly increased when ammonium concentration increased from 2 to 10 ppm, suggesting that nitrifiers in this savanna soil were not adapted to ammonium levels as low as for the soil of central Ivory Coast.

Even after 5 crop cycles, *i.e*. after 2.5 years, nitrification was not influenced by the cropping systems, including mulching and the insertion of legumes in the crop rotation. The lack of response of nitrification to agricultural practices is particularly striking for the fMM treatment that implied the addition of 25 g N m^−2^ per crop cycle as urea and ammonium. This result is not consistent with many previous studies which showed that nitrification is stimulated by chemical fertilization in many cropland and grassland ecosystems^53, 54^ (see the synthesis of previous reports in Fig. 2).

Over the same 2.5 year period, changes in the abundances of some nitrifier groups were observed, particularly at the end of the study period. The abundance of AOB increased in response to fertilization after 5 crop cycles while the abundance of AOA was not influenced by the fMM treatment. This result is consistent with previous studies reporting an increase of AOB rather than AOA abundance in response to chemical fertilization through the addition of NPK and/or urea to soil^19, 55^. In addition, many studies have observed increased abundance and activity of AOB in response to N fertilization^56^ and other studies have showed that the abundance of AOA remains unaffected or even decreases in response to N addition in grasslands^17, 19^ and other temperate ecosystems^18^. Recently, Catão *et al*.^57^ reported that the AOB-to-AOA ratio as nitrification increased along different stages of soybean cultivation in a site under restoration from gravel extraction in the Central Brazilian savanna zone. All these results are likely linked to the rough niche partitioning between AOB and AOA regarding N availability in soil^14^. In our study, we also observed that *Nitrobacter* and *Nitrospira* abundances increased and were not changed in response to fertilization, respectively. These findings are consistent with some published results for NOB dynamics in agricultural and forest soils^6, 30^. Attard *et al*.^6^ and Le Roux *et al*.^29^ found that *Nitrobacter* are favored under high N availability while *Nitrospira* are less responsive to N level in agricultural and grassland soils. Overall, our results support the view that AOB and *Nitrobacter* are the nitrifying groups mostly stimulated by an increase in N availability, while AOA and *Nitrospira* abundances are not much responsive to increased N availability, as observed for other grassland soils^19^.

The overall AOB community structure was not influenced by treatment and was mainly related to soil moisture, which is likely due to the fact that our experimental design included 9 sites located across a landscape. Despite this fact, several groups of AOB were selected in the different fertilized plots. In particular, we identified 17 clusters of AOB-*amoA* sequences whose relative abundances increased in response to fertilization as compared to control soils. Because soil pH and soil water content were not affected by the different cropping treatments, N fertilization *per se* was likely the main driver of the treatment-induced selection of these AOB populations. Two facts support this view. First, several *amoA* sequences that emerged in fertilized plots were affiliated to *amoA* sequences of *Nitrosospira* strains with urease activity or adapted to fluctuating ammonium levels. In particular, some AOB favoured by fertilization were affiliated to *Nitrosospira multiformis* whose genome includes (i) several copies of both *amo* and *hao* gene clusters and their regulatory elements that extend the flexibility for expression of catabolic inventory under fluctuating ammonia concentrations; (ii) a range of genes allowing ammonia assimilation either by high-affinity systems when ammonium concentration is low, or a low-affinity system used when ammonium levels are high; and (iii) several gene clusters encoding urease and urea-carboxylase, which provides ureolytic capacity adaptative for soils experiencing fluctuating urea concentrations and/or acidic pH^58^. Furthermore, other AOB favoured by fertilization were affiliated to *Nitrosospira briensis* which is known for its survival strategy under fluctuating ammonium availability, involving low decrease of ammonia oxidation activity following inception of N starvation, and fast recovery after N addition following a starvation period^59^. This can be explained by the forms of N fertilizer (ammonium or urea) added to the soil 10 times during the 2.5 years period studied, which has likely induced transient increases of urea and/or ammonium levels in soil and thus strongly selected AOB able to efficiently use these substrates even at relatively high levels and to cope with strong fluctuations of urea/ammonium levels. The likely transient nature of N increases in fertilized plots, explained by the very low cation exchange capacity of these soils and high rainfall amount, could also explain why the shift of the AOB community was slow. Second, considering all the treatments, the relative abundance of AOB populations that emerged in fertilized plots was correlated to the nitrifying community ability to maintain its activity at medium (10 ppm) as compared to low (2 ppm) ammonium levels. This is a further indication that these AOB populations had ecophysiological traits allowing them to withstand increased ammonium levels. Metagenomics analyses could allow better description of the genome content of these populations.

Our results thus suggest that, while populations dominating the AOB community in control soils were adapted to very low N levels, niche differentiation among AOB (possibly NOB as well) linked to their responses to fluctuating N availability and starvation responses (see refs 60, 61) explained the emergence of new dominant populations in fertilized plots. The changes in the composition of the nitrifier community required for adaption to higher –and fluctuating– N levels occurred slowly. In the case of the mulching practice with maize-bean and moreover maize-soya rotation, the changes were even slower than with chemical fertilization. The N-related community composition changes can also explain why the correlations between the abundances of the different nitrifier groups changed with time. After 3 crop cycles, the increase of both AOB and *Nitrobacter* abundances and the selection of nitrifier populations in response to fertilization events were likely still not effective enough and no significant correlation was observed between *Nitrobacter* and AOB abundances. In contrast, after 5 crop cycles, our results show that both the increase in total AOB and *Nitrobacter* abundances and the selection of AOB (and likely *Nitrobacter*) populations were effective; consistently, at this date the correlation between *Nitrobacter* and AOB abundances became significant. Our results thus strongly suggest that the response of the nitrifying community to fertilization is mainly driven by the increased fitness of some AOB, and likely some *Nitrobacter*, able to benefit from fertilization event, while AOA and *Nitrospira* populations are not (or only marginally) responsive to increased N availability.

### Conclusion: from the ecology of soil nitrifiers to the choice of economically-sound and sustainable agronomic practices

In this study, we showed that among the key microbial activities linked to N cycling (such as N_2_ fixation, mineralisation, nitrification, denitrification and anaerobic ammonium oxidation^62^), nitrification plays a crucial role in determining the outcome of agronomic practices. Indeed nitrifiers in soils from the moist savanna zone of Ivory Coast are adapted to very low N level, medium ammonium levels even decreasing soil nitrification rates as compared to low ammonium levels. Dominant nitrifiers in these soils are actually unable to benefit from sudden increases in N availability induced by recurrent fertilization events. Because the maize cultivar used by farmers in the area studied has a strong preference for nitrate over ammonium for its N nutrition, the lock-up of the N cycle by nitrifiers, which restricted the conversion of the added urea and ammonium into nitrate, jeopardized the efficiency of chemical fertilization, at least during 2–3 years. This is witnessed by the lack of improvement of the maize N status in fertilized plots even after 2.5 years. As indicated above, chemical fertilization likely induced fluctuating N concentrations, with high ammonium and urea levels just after fertilization alternating with periods of low levels. Accordingly, a deep change in the AOB community composition, with the selection of taxa able to cope with fluctuating N levels and/or able to use urea as a N source, was first needed before nitrification could increase in response to N addition. A time lag in nitrification response to fertilization due to inadequate nitrifier community composition has already been reported for other soils, but it is much longer for the moist savanna soils where nitrifiers are adapted to particularly low N level. Our results show that the ecology of nitrifiers and the poor N retention capacity in these soils experiencing a sub-equatorial climate must be taken into account to guide the choice of agricultural practices, explaining why chemical fertilization is not a smart solution for cropping in the moist savanna zone.

## Materials and Methods

### Study site and experimental design

This study was conducted in the moist savanna zone, in the surroundings of the villages of Aheremou 2 and Pacobo in Ivory Coast (see Fig. S1). The area has a sub-equatorial climate with a mean annual temperature of 27 °C and mean annual precipitation of 1200 mm. In this area, soils are Ferralsol (FAO classification) or Acrisols (World Reference Base - IUSS Working group). They are very sandy (80–90% sand) and characterized by pH from 6 to 7^63^, with low organic matter (~1%) and nitrogen ( < 0.1%) contents in non cultivated areas. Clays are illites and slightly crystallized kaolinites with a low adsorption capacity^42^. The traditional agricultural practice in the area is slash-and-burn, with typically 2 crop cycles per year.

Nine experimental sites (19 m × 12 m) were established in early July 2013 across the landscape (see Figs S1 and S2). All the experimental sites correspond to moist savanna soils used for local agriculture. Before the experiment inception, all sites first experienced a succession of yam, banana and cassava crops without fertilization and were then set aside: they thus were all fallows invaded by *Chromolaena odorata* when the treatments were applied. At each site, 6 plots (4 m × 5 m each) were set up, corresponding to 6 different agricultural practices for maize (M) cultivation: (1) traditional slash-and-burn practice with continuous maize rotation (sMM) where the biomass present on the plot before planting (i.e. *Chromolaena odorata* residues at the beginning of cultivation of fallows; and then aboveground residues of the previous crop) is burned and ashes spread over the plot; (2) use of chemical fertilizer with continuous maize rotation (fMM), with addition of NPK (corresponding to 15g N-ammonium m^−2^) at planting, plus urea application (10g N-urea m^−2^) 40 days after planting (note that we analysed a sample of the fertilizer used to check its nature and concentration); (3) mulching technique with continuous maize rotation (mMM) where the biomass present on the plot before planting is spread at the soil surface over the plot; (4) mulching technique with maize-soya rotation (mMS); (5) mulching technique with maize-bean rotation (mMB); and (6) bare soil with continuous maize rotation (bMM) which was used as a control without any input to soil. All plots were thus planted with maize, except mMS and mMB planted with soya and bean during the second, third and fifth cyles. The plots were weeded prior to sowing. The maize variety used is a cross between the yellow maize type ‘Bouake’ and the white corn type ‘IRAT 8’ (EXECO Agriculture, Abidjan, Ivory Coast). It is an open-pollinated variety with a semi-late cycle of 110–130 days, widely used in this area.

### Soil sampling

Soil was sampled on the 54 plots (i.e. 9 sites of 19 m × 12 m with 6 treatments per site) 3 times at flowering crop stage: on December 2014, before the end of the third crop cycle at the beginning of dry season (i.e. 18 months after treatment inception); on June 2015, before the end of the fourth crop cycle in the middle of wet season (after 24 months); and on December 2015, before the end of the fifth crop cycle at the beginning of dry season (after 30 months). On each plot, soil sub-samples were collected randomly at 5 distinct points using an auger (0–10 cm; 5 cm diameter) and mixed to obtain a composite sample. Composite samples were packed in plastic bags and transported in an icebox to the laboratory. Each soil sample was sieved (2mm) and a sub-sample was used for measurements of nitrification activity and other environmental variables. Another sub-sample was stored at −20 °C before DNA extraction and measurement of nitrifier abundances and AOB diversity.

### Measurements of nitrification activity

Nitrification activity was measured according to Niboyet *et al*.^64^ as the kinetics of production of nitrite plus nitrate measured after 0, 5, 24, 48 and 72 hours of incubation in aerobic conditions at 28 °C. For each of the 54 soil samples at each date, a fresh soil sub-sample (3 g equivalent dry mass of soil) was placed in a 150 mL flask, and 24 ml of a (NH_4_)_2_SO_4_-distilled water solution was added (2 µg N g^−1^ soil). According to preliminary analyses performed on some soils for different treatments after 3 crop cycles, the amount of added ammonium was identified as that allowing the maximum nitrification rate in control soils.

For the last date only, nitrification activity was also measured for each of the 54 samples at a higher ammonium level. Incubation conditions were similar as above, except that the amount of (NH_4_)_2_SO_4_ added to the soil was 5-fold higher, i.e. 10 µg N- NH_4_^+^ g^−1^ soil.

### Comparison of the response of nitrification to soil ammonium concentration for other soils

The response of nitrification to soil ammonium concentration observed for the soil studied here was compared with the responses observed for a temperate and fertilized cropland soil from central France (4 independent samples collected on Lusignan site; soil characteristics provided in Attard *et al*.^65^) and for a non fertilized and non cropped mesic savanna soil from South Africa (4 independent samples collected on Mona site, Hluhluwe Imfolozi National Park). For each sample of the fertilized cropland soil, seven ammonium levels were used, *i.e*. addition of 0, 5, 20, 50, 100, 200 or 300 µg N-NH_4_^+^ g^−1^ soil. For each sample of the South African savanna soil, three ammonium levels were used, i.e. 2, 5 or 10 µg N-NH_4_^+^ g^−1^ soil. Nitrification rate was measured as detailed above.

### Comparison of N fertilization effects on nitrification reported for cropping and grassland systems

A literature survey was performed in order to compare our results to the effects of long-term fertilization on nitrification in cropping and grassland systems. We focused on fertilization with mineral N and/or urea (Table S1), but excluded studies on the effect of other organic fertilizers. For each study (and each fertilizer level when relevant), the fertilization effect on nitrification was expressed as the percentage of change regarding the nitrification level measured in control, unfertilized plots.

### DNA extraction and quantification of the abundances of ammonia- and nitrite-oxidizers

DNA was extracted from 0.5 g of frozen sieved soil using the Power Soil TM DNA Isolation Kit (MO BIO laboratories, Carlsbad, CA, USA) following the manufacturer’s instructions.

The abundances of ammonia oxidizing archaea and bacteria (AOA and AOB, respectively) were measured by quantitative PCR targeting the *amoA* functional gene encoding for ammonia monooxygenase which is specific of these groups. Amplification was performed using gene primers CrenamoA23f (5′-ATGGTCTGGCTWAGACG-3′) and CrenamoA616r (5′-GCCATCCABCKRTANGTCCA-3′) for the AOA^66^ and amoA_1 F (5′-GGGGHTTYTACTGGTGGT-3′) and amoA_2 R (5′-CCCCTCKGSAAAGCCTTCTTC-3′) for the AOB^67^ (see details about qPCR conditions in Supplementary Text S1).

The abundance of *Nitrobacter*-like NOB was measured by quantitative PCR targeting the functional gene *nxrA* according to Attard *et al*.^5^, this gene encoding for the nitrite oxido-reductase. The amplification was performed using the gene primers F1norA (5′-CAGACCGACGTGTGCGAAAG-3′) and R2norA (5′-TCCACAAGGAACGGAAGGTC-3′)^68^ (see details about qPCR conditions in Supplementary Text S1).

The abundance of *Nitrospira* was measured by quantitative PCR targeting the *16S* rRNA gene sequences specific for this group according to Attard *et al*.^5^. The amplification was performed using the gene primers Ns675f (5′-GCGGTGAAATGCGTAGAKATCG-3′) and Ns646r (5′-TCAGCGTCAGRWAYGTTCCAGAG-3′)^69^.

For all 4 genes, amplification efficiencies were of 85–90%. The R^2^ values of the standard curves for qPCR assays were always over 0.99. All microbial abundances were expressed on a dry soil basis.

### Assessment of AOB diversity

High-throughput sequencing of AOB *amoA* genes were performed on an Illumina MiSeq® platform by Molecular Research DNA, USA for the 54 samples from the last sampling date (i.e. after 5 crop cycles). A combination of the tools available from the RDP FunGene website^70^ and the open-source software Mothur (v.1.33.3)^71^ was used to process and analyze the sequence data.

Sequencing products were first paired in overlapping pair ends. Resulting sequence data were then sorted according to their length, and the quality of the primers (<2 errors) and barcodes (<1 error). The primers and barcodes were trimmed off before searching potential chimeric formation using UCHIME^72^ implemented in Mothur. Putative chimeras were removed from the dataset. *AmoA* nucleotide sequences were translated in amino-acids, and possible frame-reading shifts were detected and corrected using the FRAMEBOT algorithm^73^. *AmoA* sequences were clustered into operational taxonomic units (OTUs) by setting a 0.05 distance limit^74^. Similarity between the *amoA* sequences retrieved from soils and reference sequences was assessed using 15,212 sequences of AOB *amoA* extracted from the FunGene database. For each sample, rarefaction curves based on identified OTUs and species richness estimator ACE were generated using Mothur.

### Measurements of soil environmental variables

For each of the 54 soil samples and at each date, gravimetric soil moisture was determined for 3 g of fresh soil from soil mass loss after drying for 24 h at 105 °C. Ammonium (NH_4_^+^) was extracted from soil sub-samples using a solution of calcium chloride (10 mM), 12 ml of CaCl_2_ being added to 3 g of equivalent dry mass soil. The soil was shaken at 140 rpm for 2 h at 10 °C. The suspensions were filtered and stored at −20 °C before measurement of the amounts of NH_4_^+^ by ionic chromatography (Thermo Scientific™ Dionex™ ICS-900 passeur AS, France). Due to a technical problem, soil nitrate (NO_3_^−^) was quantified only for the last sampling date, i.e. after 5 crop cycles, using the same method but with a specific column for the chromatagraph. Soil pH was measured at each date in a soil:water (1:2.5) suspension using glass electrodes (sensION^+^ MM 340 HACH, France).

In addition, at the first and last sampling dates (after 3 and 5 crop cycles), soil organic C, total soil nitrogen and microbial biomass were determined. Soil organic carbon was determined by the partial oxidation method^75^ through titration against 1 N (NH4)_2_ Fe (SO4)_2_.6H_2_O using diphenylamine indicator. Total soil nitrogen was quantified using the Kjeldahl digestion method^76^. A proxy of soil microbial biomass, dehydrogenase activity, was determined on 1 g eq. dry soil by reduction of Triphenyl Tetrazolium Chloride to Triphenylformazan as described by Thalman^77^.

### Characterization of preferred mineral N forms up-taken by the maize variety, and N concentrations in plant compartments

20 pots (10 cm diameter × 21.5 cm depth) containing 1.8 kg of sand were used. 3 maize seeds were planted per pot, and plants were grown in a climatic chamber (21 °C, 16 hours of sunshine per day). During 5 weeks, the maize seedlings were watered three times per week: 2 times with 80 mL distilled water and once with 80 mL of hydroponic solution containing 6 ppm of ammonium-nitrate (equal amounts of both N forms).

After 5 weeks, the roots of each set of three plants (i.e. from each pot) were gently washed with distilled water and placed in a 0.5 L flask filled with a nutrient solution with a 5:1 nitrate:ammonium ratio. Two concentrations of total mineral N were used: 6 ppm mineral N (5 ppm N-NO_3_^−^ and 1 ppm N-NH_4_^+^), and 17 ppm mineral N (14.15 ppm N-NO_3_^−^ and 2.83 ppm N-NH_4_^+^). 5 replicates (pots) were used per treatment. For each flask, the solution was sampled with a syringe (1.5 mL) at the beginning of experiment and after 25, 50, 75 and 100 min. Samples were filtered (25 μm) and stored at −20 °C before analysis by ionic chromatography (Thermo Scientific™ Dionex™ ICS-900, France). The rates of absorption of ammonium and nitrate by roots were computed from the linear decreases of ammonium and nitrate concentrations in the solution with time, and were expressed as mg-N h^−1^ g^−1^ dry maize root.

For the last sampling date, N concentrations in plant compartments (roots, stems and leaves) were measured as described in Supplementary Text S2.

### Statistical analyses

All statistical analyses were conducted with JMP® Pro software (version 12, SAS Institute Inc., Cary, NC, 2007). Two-Way ANOVAs were carried out for evaluating possible effects of time, treatment and time X treatment on environmental variables (soil moisture, pH, ammonium, soil organic C, total soil N and dehydrogenase activity), nitrification activity, and abundances of AOB, AOA, *Nitrobacter* and *Nitrospira*. For each date, One-Way ANOVAs were carried out to test for possible treatment effects on these variables and absorption of ammonium and nitrate by roots, and Tukey HSD tests were applied for mean comparisons between treatment pairs. Significant differences were considered for p < 0.05.

The AOB community structure was analysed using the PRIMER software (PRIMER-E Ltd, Plymouth, UK). A rank similarity matrix was computed from the OTU relative abundance data and used to quantify and visualize the dissimilarity of community structures among soil samples by non-metric multidimensional scaling (MDS). Analysis of similarities (ANOSIM) was performed to compare the AOB community structures among each pair of treatments. The correlation between changes in nitrification and changes in the overall AOB community structure was tested by quantifying the correlation between the rank similarity matrices obtained for AOB community structure on the one hand and nitrification on the other hand through the computation of the rank correlation coefficient (Spearman coefficient) and significance level. A similar approach was used to test the relationships between changes in the overall AOB community structure and changes in each of the soil environmental parameters measured. In addition, based on the phylogenetic tree of *amoA* sequences retrieved from all soils, we identified which *amoA* sequence clusters showed an increased relative abundance in fertilized as compared to control plots.

For the last sampling date, the linear correlation between (i) the percentage of change of nitrification measured for 10 ppm as compared to 2 ppm ammonium (i.e. 100 * (Nitrif_10_ − Nitrif_2_)/Nitrif_2_) and (ii) the AOB/AOA ratio or the *Nitrobacter*/*Nitrospira* ratio was tested. In addition, the percentage of change of nitrification measured for 10 ppm as compared to 2 ppm ammonium was correlated to the sum of the relative abundances of these *amoA* OTUs promoted by fertilization.

## Electronic supplementary material

Supplementary Material
