# Supplementary material for: Adaptation of soil nitrifiers to very low nitrogen level jeopardizes the efficiency of chemical fertilization in west african moist savannas
Source: Sci Rep. 2017 Aug 31;7:10275. doi: 10.1038/s41598-017-10185-5 (PMC5578973; doi:10.1038/s41598-017-10185-5)
Supplement: Supplementary file 1 — Supplementary Material [file 41598_2017_10185_MOESM1_ESM.pdf]

Supplementary Material – *Scientific Reports*

Adaptation of soil nitrifiers to very low nitrogen level jeopardizes the efficiency of chemical fertilization in West African moist savannas

by

Assémien L. Féline, Pommier Thomas, Gonnety T. Jean, Gervaix Jonathan & Le Roux Xavier

Supplementary text

Text S1: qPCR conditions

For ammonia oxidizers, the final reaction volume was 20 µl and contained (final concentration) 0.75µM of CrenamoA616r and 1µM of CrenamoA23f for the archaeal amoA or 0.5 µM of each primer for the bacterial amoA, 2 % bovine serum albumin (BSA), and 1× of Quanti Tect SybrGreen PCR Master Mix (Qiagen, Courtaboeuf, France). Samples were analyzed in duplicate on a lightcycler 480 (Roche Diagnostics, Meyland, France). For AOA, PCR cycles were as follows: at 95°C for 15 minutes, 45 amplification cycles (94°C for 45 s, 55°C for 45 s and 72°C for 45 s) and 10 s at 40°C. For AOB, PCR cycles were as follows: at 95°C for 15 minutes, 45 amplification cycles (94°C for 30 s, 54°C at 45 s and 72°C at 45 s) and 15 s at 80°C. Standard curves were developed by serial dilution of a plasmid harbouring both archaeal 54d9 and *Nitrosomonas europaea* (GenBank accession number L08050) *amoA* gene fragments to the final concentrations of 10^7 to 10^1 gene copies μL^{-1}.

For *Nitrobacter*, the final reaction volume was 20 µl and contained (final concentration) 0.5 µM of each primer, 1× of Quanti Tect SybrGreen PCR Master Mix (Qiagen, Courtaboeuf, France), and 40 ng of soil DNA extract or 10^7–10^2 copies using a linearized plasmid containing cloned *nxrA* gene of *Nitrobacter hamburgensis* X14 (DSMZ 10229). The samples

were run twice on a Lightcycler 480 as follows: 15 min at 95°C and 45 amplification cycles (30 s at 95°C, 45 s at 55°C, and 45 s at 72°C).

For *Nitrospira*, the final reaction volume was 25 µl and contained (final concentration) 0.4 µM of each primer, 1× of QuantiTect SybrGreen PCR Master Mix (Qiagen, Courtaboeuf, France), and 10 ng of soil DNA extract or 10^7–10^2 *Nitrospira-16S* copies inserted in a linearized plasmid DNA (GenBank accession number: FJ529918). qPCRs were run twice on a Lightcycler 480 as follows: 15 min at 95 °C and 45 amplification cycles (30 s at 95°C, 30 s at 66°C, and 1 min at 72°C).

Text S2: Measurements of nitrogen concentration in crop leaves, stems and roots.

For the third sampling date, i.e. at the end of the fifth crop cycle, three plants of maize (or beans and soya for mMB and mMS, respectively) were collected on 18 of the 54 experimental plots (i.e. on 3 of the 9 sites randomly selected). Plant compartments (roots, stems and leaves) were sorted and dried at 45 ° C in a ventilated oven (New Brunswick Scientific, EDISON, USA) for one week. Dried plant compartments were ground to obtain a fine powder using a TissueLyser II bead mill (Qiagen, France) with metal beads. 3 mg of each sample was placed into 5 x 8-mm “Ultra Clean” tin capsules (Elemental Microanalysis, UK) and submitted for IRMS (Isoprime 100, Isoprime Ltd, UK) coupled with an elemental analyser (Thermo FlashEA 1112, ThermoElectron, USA) to determine its N concentration.

Supplementary Figure S1

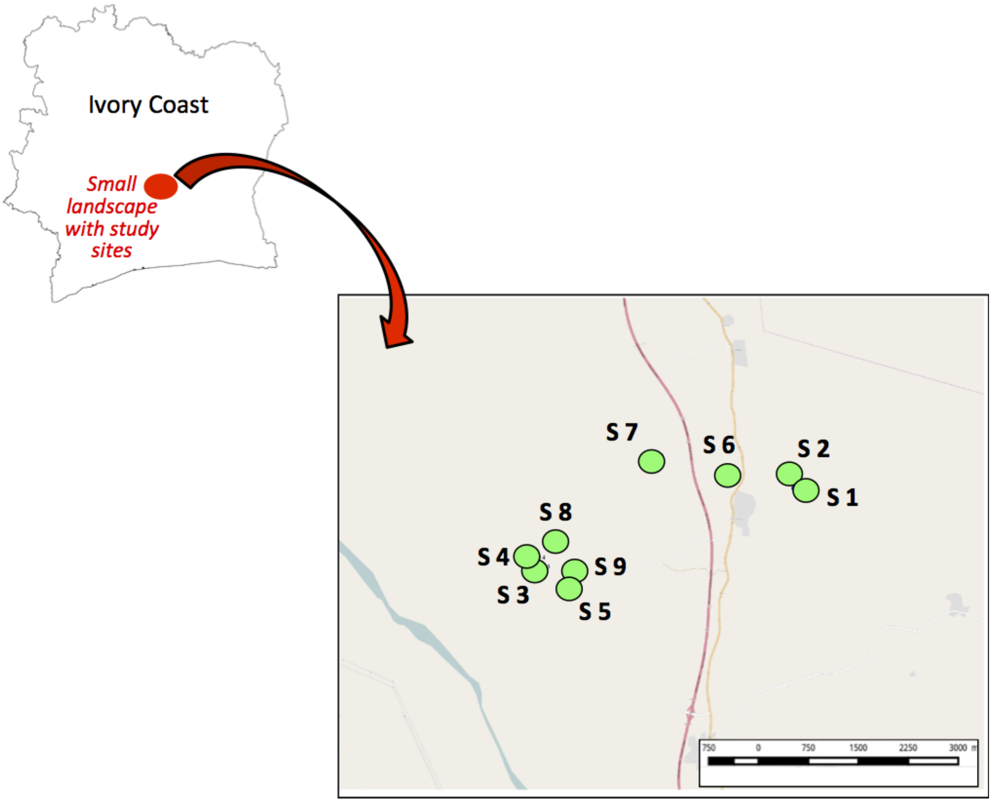

Sites	Location		
	**Longitude**	**Latitude**	**Altitude (m asl)**
S 1	06º13’28.6"	004º54’58.9’’	89
S 2	06º13’37.6’’	004º55’09.5’’	98
S 3	06º12’26.3’’	004º58’47.5’’	113
S 4	06º12’29.5’’	004º58’51.0’’	113
S 5	06º12’10.4’’	004º58’21.6’’	137
S 6	06º13’37.4’’	004º56’01.1’’	104
S 7	06º13’49.9’’	004º57’08.8’’	112
S 8	06º12’42.1’’	004º58’30.6’’	135
S 9	06º12’18.1’’	004º58’15.7’’	133

Fig. S1. (Top) Location of the small landscape including the study sites in Ivory Coast, and location of the 9 study sites (green dots) across this landscape. The Ivory Coast and landscape maps were drawn by the authors (i.e. with changes made) respectively based on the Global Administrative Areas (2012 - GADM database of Global Administrative Areas, version 2.0. [online] URL: www.gadm.org) and on OpenStreetMap (www.openstreetmap.org, which is open data, licensed under the Open Data Commons Open Database License, while the cartography in the OpenStreetMap map tiles is licensed under CC BY-SA – see www.openstreetmap.org/copyright). (Bottom) GPS coordinates and altitude of each study site.

Supplementary Figure S2

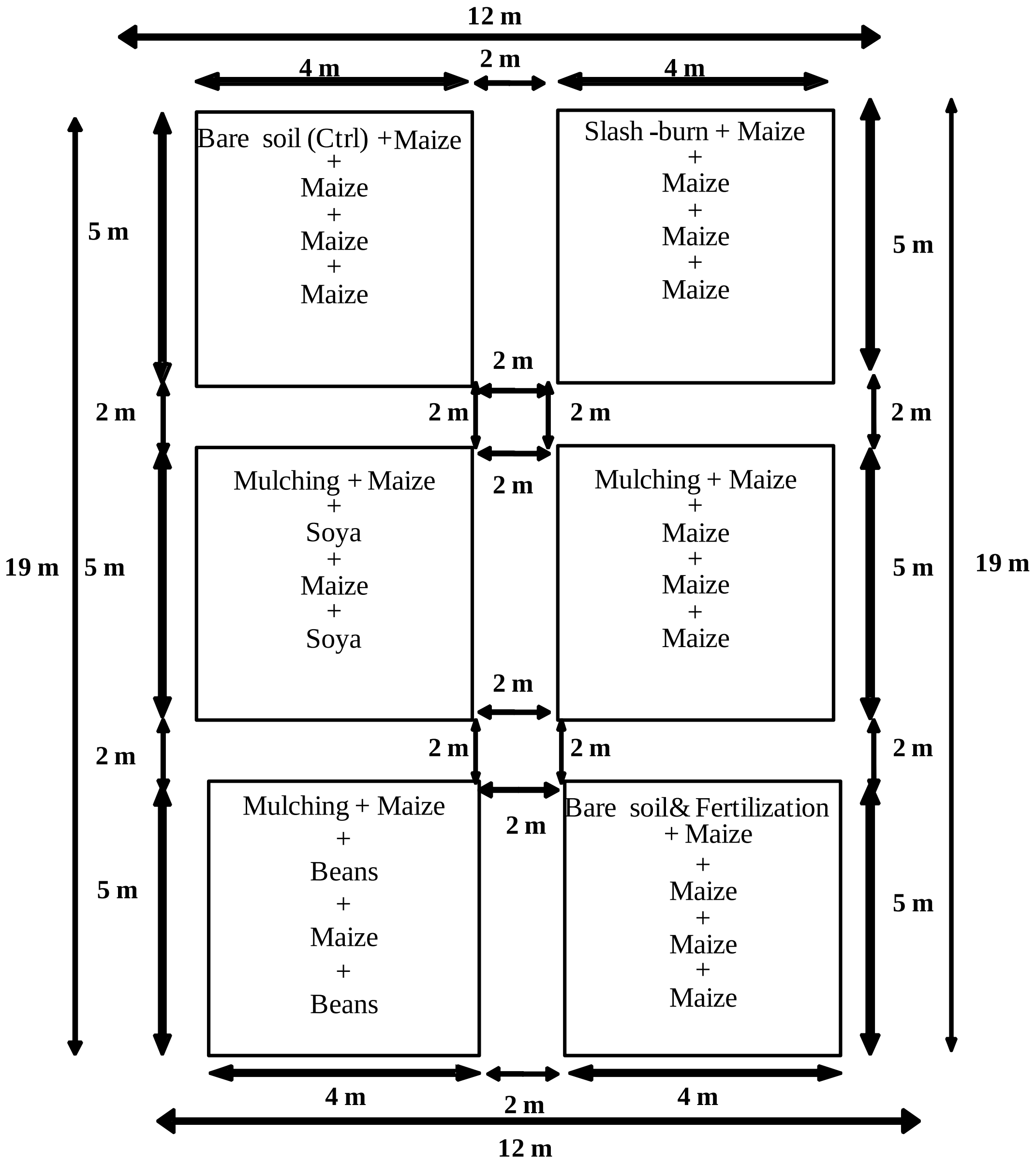

Fig. S2. Experimental design used at each of the 9 study sites. Six treatments were studied: bMM, maize-maize rotation on bare soil; sMM, maize-maize rotation with the slash-and-burn practice; mMM, maize-maize rotation with the mulching practice; mMB, maize-bean rotation with the mulching practice; mMS, maize-soya rotation with the mulching practice; fMM, maize-maize rotation with chemical fertilization.

Supplementary Figure S3

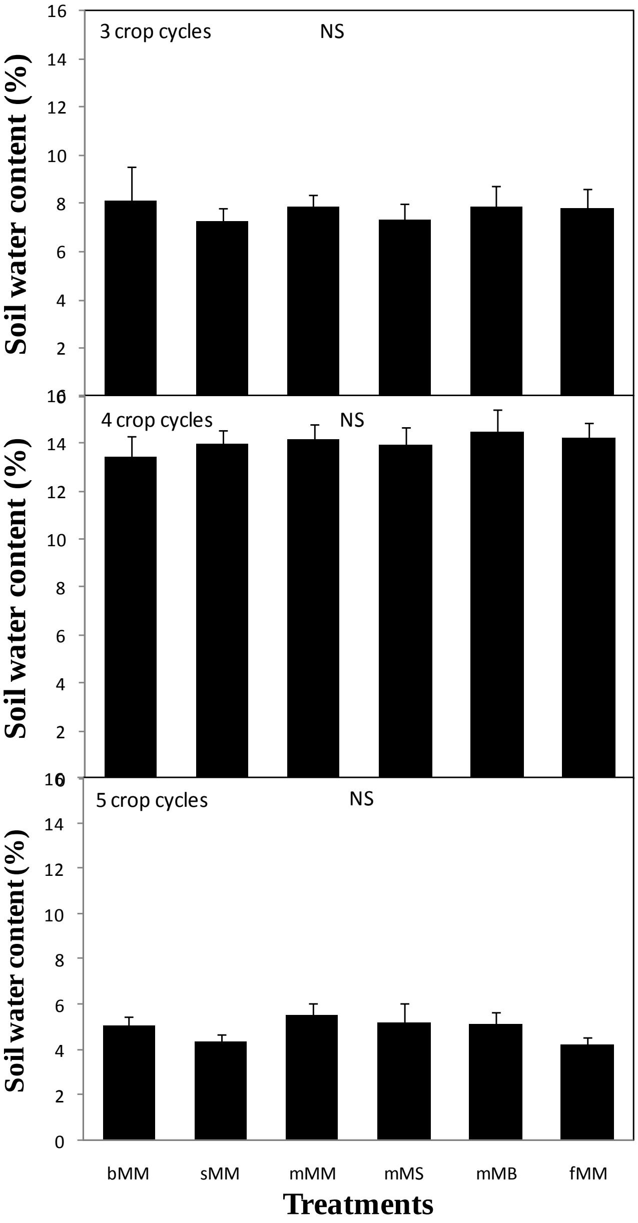

Fig. S3. Gravimetric soil water content according to treatment after 3, 4 and 5 crop cycles. Treatment acronyms are as in Fig. S2. Error bars are standard errors (n=9). NS: no significant difference between treatments.

Supplementary Figure S4

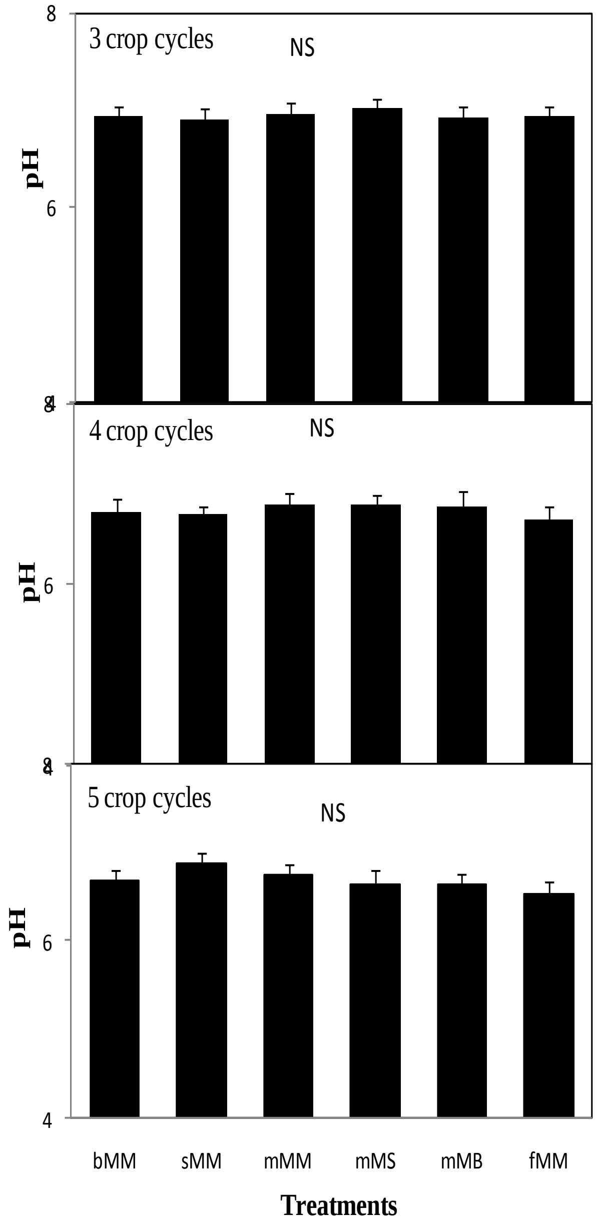

Fig. S4. Soil pH according to treatment after 3, 4 and 5 crop cycles. Treatment acronyms are as in Fig. S2. Error bars are standard errors (n=9). NS: no significant difference between treatments.

Supplementary Figure S5

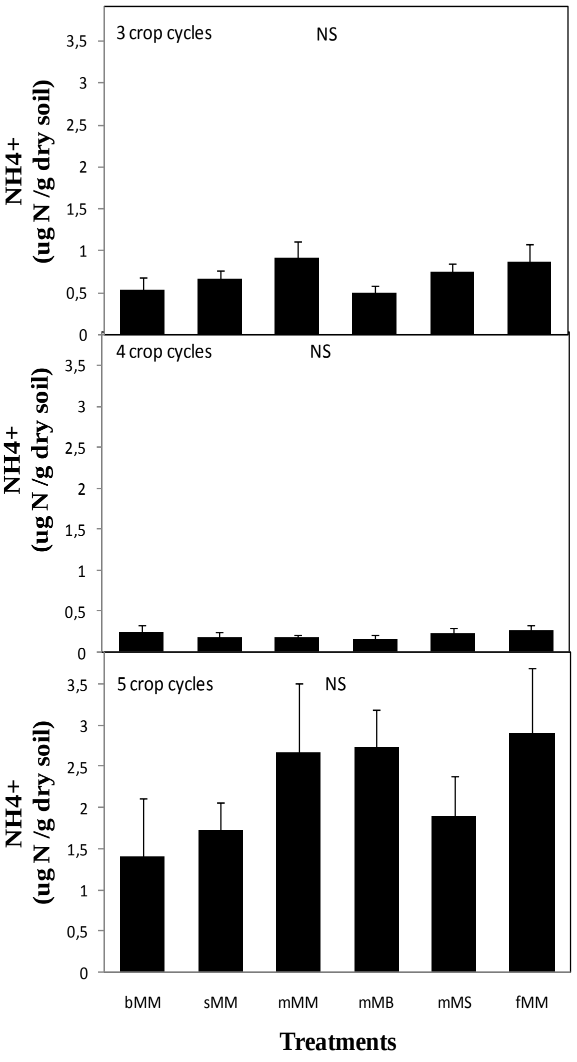

Fig. S5. Soil ammonium concentration according to treatment after 3, 4 and 5 crop cycles. Treatment acronyms are as in Fig. S2. Error bars are standard errors (n=9). NS indicates lack of significant difference.

Supplementary Figure S6

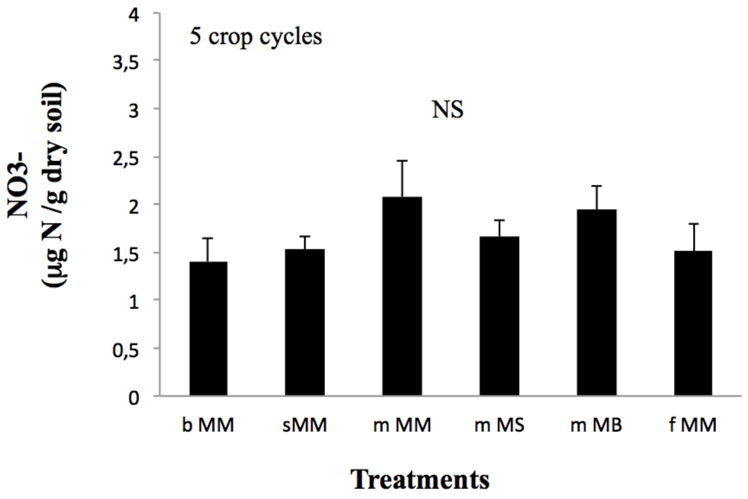

Fig. S6. Soil nitrate concentration according to treatment measured after 5 crop cycles. Treatment acronyms are as in Fig. S2. Error bars are standard errors (n=9). NS indicates lack of significant difference.

Supplementary Figure S7

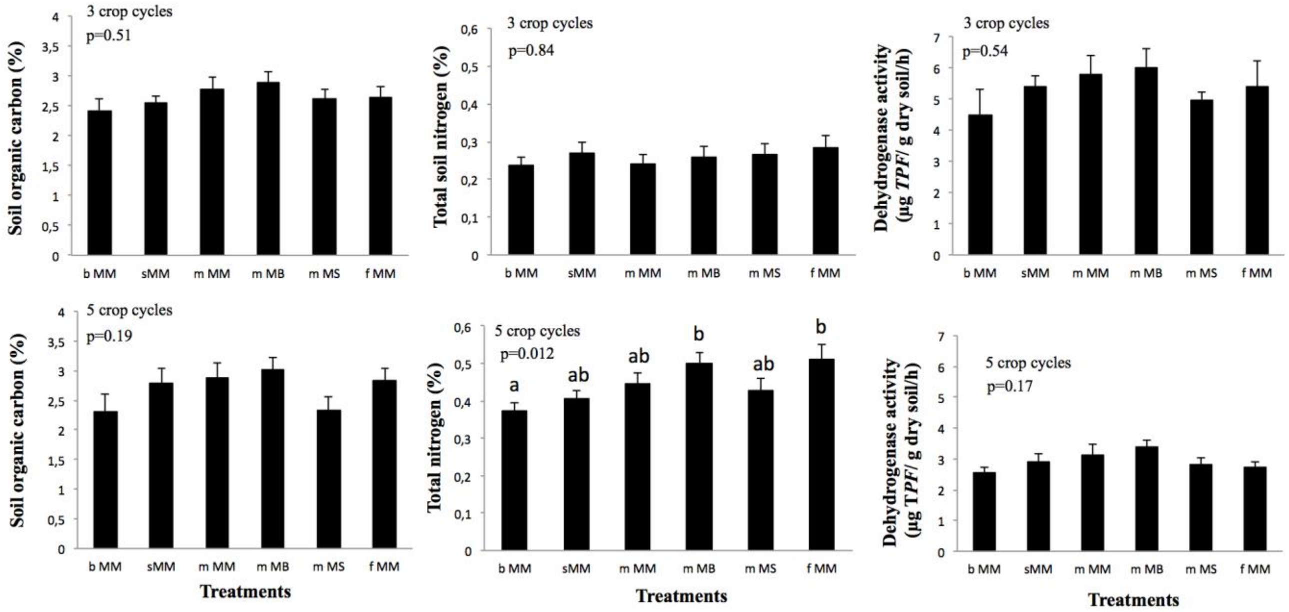

Fig. S7. (Left) Soil organic carbon concentration, (Middle column) total soil nitrogen concentration, TN, and (Right) a proxy of microbial biomass, i.e. dehydrogenase activity, according to treatment after (Top) 3 crop cycles and (Bottom) 5 crop cycles. Treatment acronyms are as in Fig. S2. Error bars are standard errors (n=9). When ANOVA indicates significant treatment effect ($p<0.05$), different letters identify significant differences.

Supplementary Figure S8

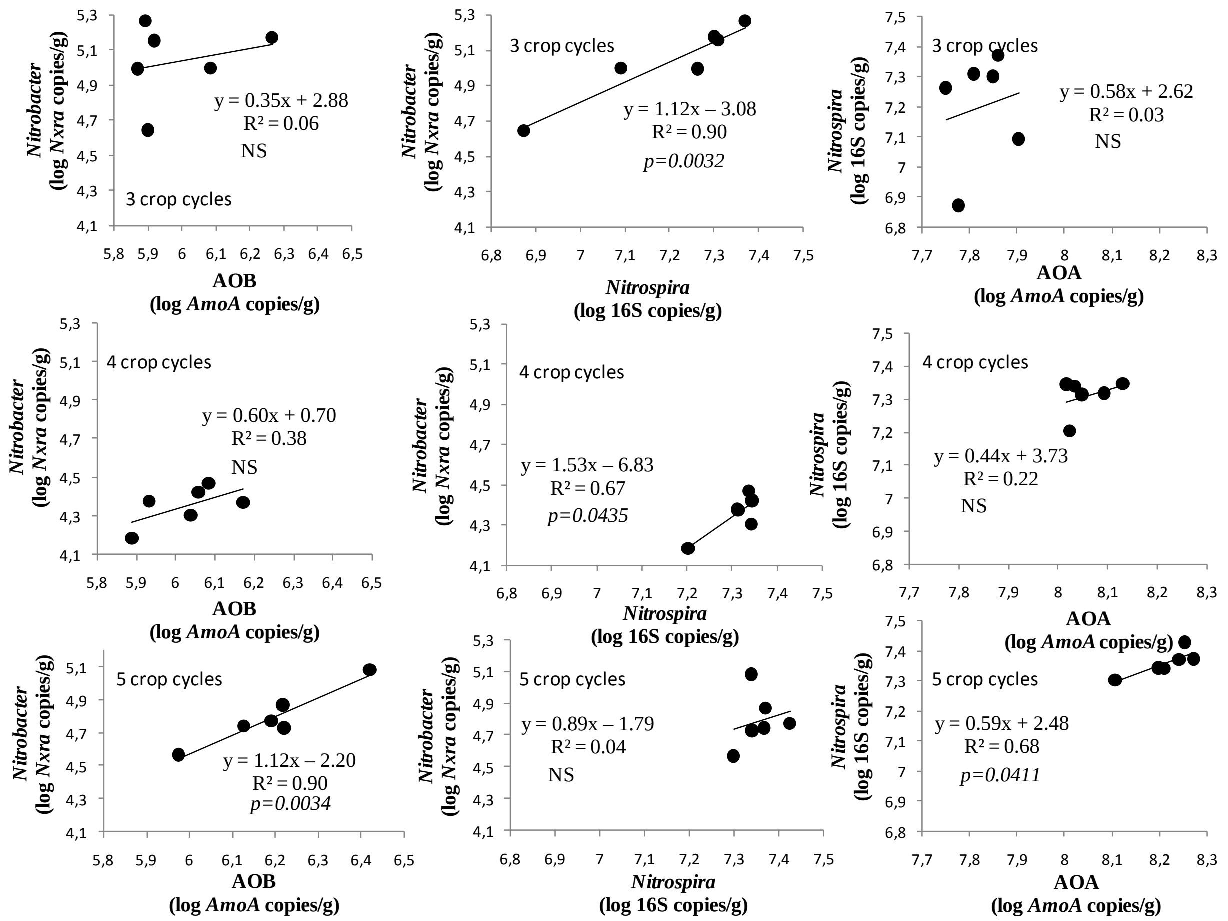

Fig. S8. Correlations between the abundances of nitrifier groups. Correlations are presented for (Left column) *Nitrobacter* and ammonia oxidizing bacteria, AOB, (Middle column) *Nitrobacter* and *Nitrospira*, and (Right column) *Nitrospira* and ammonia oxidizing archaea, AOA, for (Top line) the third crop cycle, (Middle line) the fourth crop cycle, and (Bottom line) the fifth crop cycle. Each point corresponds to a treatment (n=9). R^2 and p values are indicated. NS indicates lack of significant correlation.

Supplementary Figure S9

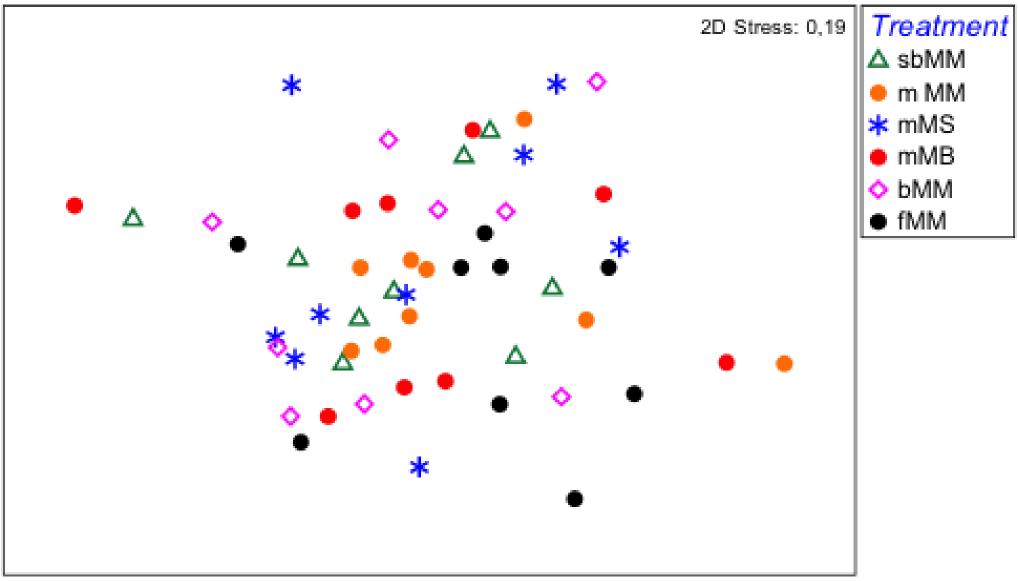

Fig. S9. Non-metric MDS ordinations of the genetic structures of the ammonia oxidizing bacteria community, for the soils from the different treatments. Treatment acronyms are as in Fig. S2. Treatment effect is not significant (p=0.93).

Supplementary Figure S10

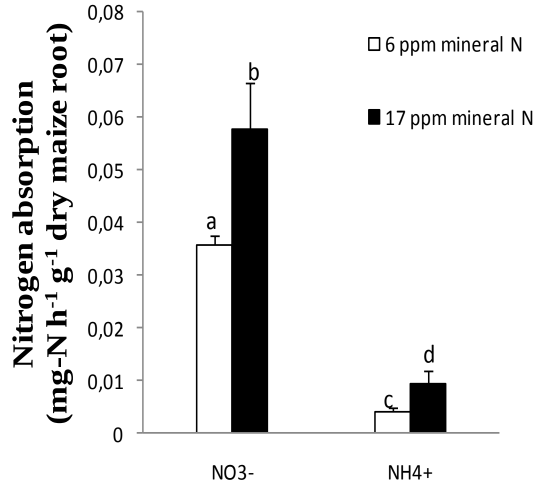

Fig. S10. Nitrogen absorption rates by maize roots at 2 different total mineral N concentrations. Measurements were made on 5 week-old plants grown in a greenhouse. Error bars are standard errors (n=5). Different letters indicate significant differences between N forms of nitrogen.

Supplementary Figure S11

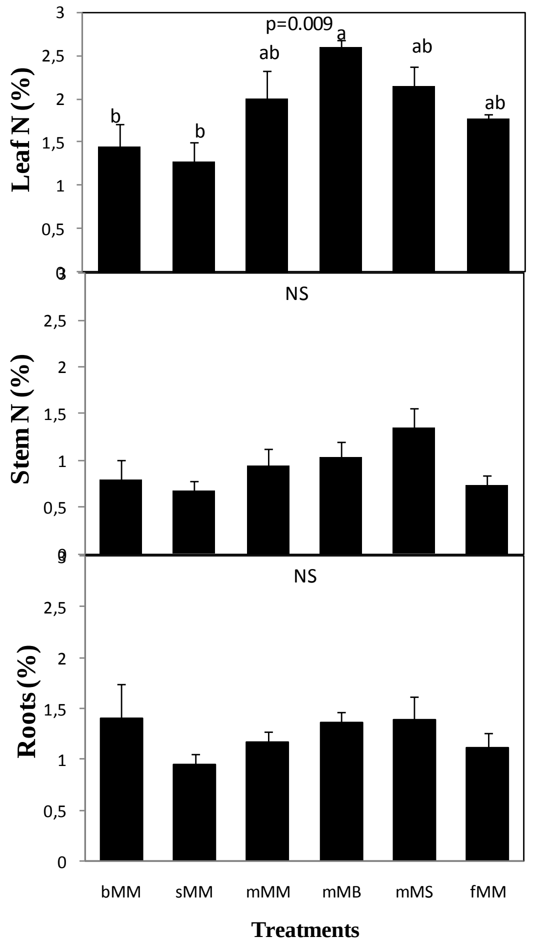

Fig. S11. Total nitrogen concentration in crop (Top) leaves, (Middle) stems, and (Bottom) roots according to treatment after 5 crop cycles. Treatment acronyms are as in Fig. S2. Error bars are standard errors (n=3). Different letters indicate significant differences between treatments; NS indicates lack of significant difference. Note that at this date, crop plants were maize in bMM, sMM, mMM and fMM plots but bean and soya in mMB and mMS plots, respectively.

Table S1. Synthesis of reported effects of long term (i.e. several years) fertilisation with mineral N and/or urea on nitrification in cropping and grassland systems. Note that several authors reported that, due to soil N turnover, even fertilisation with nitrate induces increased ammonium availability over the long term.

Ecosystem type	Fertilisation type	Fertilisation duration	Quantity of N added per growing season	% variation in nitrification	References
Moist savanna land used for agriculture (Ivory Coast)	$NH_4^+NO_3^-$ + Urea	2.5 years	25 g-N m^{-2}	-3.45%	This work
Malan Farm land for agriculture (Hebei, China)	Urea	31 years	30g-N m^{-2}	+112.5%	Ai et al. (2013)
Mediterranean grassland (California, USA)	NO_3^-	5 years	7g-N m^{-2}	+66.1%	Barnard et al. (2006)
Temperate arable land (Sweden)	NO_3^-	3 years	12g-N m^{-2}	+72.3%	Berg and Rosswall (1987)
Temperate steppe (Mongolia, China)	Urea	5 years	5g-N m^{-2}<br>15g-N m^{-2}	+182.3%<br>+205.8%	Chen et al. (2013)

Temperate grassland (France)	Urea	2 years	7g-N m^{-2}	+122.2%	Le Roux et al. (2008)
Alpine grassland (Tibetan Plateau, China)	$NH_4^+NO_3^-$	4 years	5g-N m^{-2}<br>10g-N m^{-2}<br>15g-N m^{-2}	+47.4 %<br>+118.5 %<br>+103 %	Ma et al. (2016)
Mediterranean grassland (California, USA)	NO_3^-	8 years	7g-N m^{-2}	+45.4%	Niboyet et al. (2011)
Temperate grassland (Michigan, USA)	$NH_4^+NO_3^-$	8 years	10.4g-N m^{-2}<br>12g-N m^{-2}	+5.20%<br>+113.1%	Phillips et al. (2000)
Temperate grassland (Scotland, UK)	$NH_4^+NO_3^-$	9 years	14g-N m^{-2}	+220.0%	Webster et al.(2002)
Purple soil used for agriculture (Chongqing, China)	Urea	21 years	13.5g-N m^{-2}	+92.3%	Zhou et al. (2014)

184
